# Rice panicle plasticity in Near Isogenic Lines carrying a QTL for larger panicle is genotype and environment dependent

**DOI:** 10.1186/s12284-016-0101-x

**Published:** 2016-06-02

**Authors:** Dewi Erika Adriani, Michael Dingkuhn, Audrey Dardou, Hélène Adam, Delphine Luquet, Tanguy Lafarge

**Affiliations:** CIRAD, UMR AGAP, F-34398 Montpellier, France; Faculty of Agriculture, University of Lambung Mangkurat, Banjarbaru, Indonesia; Crop and Environment Science Division (CESD), International Rice Research Institute (IRRI), Los Baños, Philippines; IRD, UMR DIADE, Montpellier, France

**Keywords:** Panicle plasticity, Panicle architecture, Branch number, Branch length, Spikelet number per panicle, Pre-floral stem vigor, *qTSN4*

## Abstract

**Background:**

Panicle architectural traits in rice (branching, rachis length, spikelet number) are established between panicle initiation and heading stages. They vary among genotypes and are prone to Genotype x Environment interactions. Together with panicle number, panicle architecture determines sink-based yield potential. Numerous studies analyzed genetic and environmental variation of plant morphology, but the plasticity of panicle structure is less well understood. This study addressed the response of rice panicle size and structure to limited light availability at plant level for near-isogenic lines (NILs) with IR64 or IRRI146 backgrounds, carrying the QTL *qTSN4* (syn. SPIKE) for large panicles. Full light and shading in the greenhouse and two population densities in the field were implemented. The image analysis tool P-TRAP was used to analyze the architecture of detached panicles.

**Results:**

The *qTSN4* increased total branch length, branching frequency and spikelet number per panicle in IRRI146 background in the field and greenhouse, and in IR64 background in the greenhouse, but not for IR64 in the field. In the field, however, *qTSN4* reduced panicle number, neutralizing any potential yield gains from panicle size. Shading during panicle development reduced spikelet and branch number but *qTSN4* mitigated partly this effect. Spikelet number over total branch length (spikelet density) was a stable allometry across genotypes and treatments with variation in spikelet number mainly due to the frequency of secondary branches. Spikelet number on the main tiller was correlated with stem growth rate during panicle development, indicating that effects on panicle size seemed related to resources available per tiller.

**Conclusions:**

The *qTSN4* effects on panicle spikelet number appear as indirect and induced by upstream effects on pre-floral assimilate resources at tiller level, as they were (1) prone to G x E interactions, (2) non-specific with respect to panicle architectural traits, and (3) associated with pre-floral stem growth rate.

**Electronic supplementary material:**

The online version of this article (doi:10.1186/s12284-016-0101-x) contains supplementary material, which is available to authorized users.

## Background

Growth of the rice plant is divided into three phases: the vegetative (from germination to panicle initiation), reproductive (from panicle initiation to heading) and grain filling or ripening phase (from heading to maturity) (Li, 1979). Yield components are progressively determined along the rice cycle, first during the vegetative phase (tiller number), second during the reproductive phase (number of fertile tillers and size of panicle sinks) and finally during the grain filling phase (spikelet filling rate). Sink-based yield potential is thus fixed by the flowering stage (Xiong et al. 2011) and depends mostly on the dimensioning of crucial organs that occurs during the reproductive phase. This is the time when the morphological structure of the panicle bearing the sinks, but also the size of internodes that store C reserves, become effective.

Panicle development consists of branching, branch elongation and spikelet deployment. These elemental processes are governed by traits that are crucial for crop breeding in getting to higher yield and improving food security (Yamagishi et al. 2004, Miura et al. 2010, Endo-Higashi and Izawa 2011, Ohsumi et al. 2011, Peng et al. 2014). At heading stage onwards, lower carbon availability to the plant like shading reduces shoot dry matter and grain filling percentage of Nipponbare rice (Kobata et al. 2000) because sink capacity is already fixed. However, during panicle development, Choi et al. (2013) reported that temporary lack of sunshine (10 – 14 days) adversely affects panicle morphogenesis (differentiated spikelet number, branch number, panicle length). According to the authors, the fact that this could be mitigated with elevated ambient [CO_2_] indicates that panicle development is driven by carbon resources although the process itself consumes little assimilate. Indeed, trait expression has to be plastic to ensure balanced source-sink relationships under variable resources. Also, Kamiji et al. (2011) reported that N top-dressing during the first stage of panicle development has a large effect on spikelet production, and genotypic differences in spikelet production could be explained by the crop growth rate during the 14-day period before heading. The sensitivity to carbon supply during this period was confirmed by Lafarge et al. (2010) on rice by looking at the detrimental effect on yield components of a 10-day shading period imposed in the field at early reproductive stage. Shiratsuchi et al. (2007) revealed that yield is correlated with the ratio of spikelet number to tiller dry weight after spikelet differentiation, and Endo-Higashi and Izawa (2011) suggested that pre-floral photosynthate accumulation determines reproductive sink capacity. This was recently confirmed by Adriani et al. (2016) analyzing near isogenic lines (NILs) with larger panicles developed by Qi et al. (2008) and Fujita et al. (2009, 2012) carrying *qTSN4* on the long arm of chromosome 4, a QTL identified for high total spikelet number (TSN). Adriani et al. (2016) reported that panicle size was strongly affected by (i) shading imposed during the reproductive phase in the greenhouse or (ii) change in plant density in the field. These authors also revealed that the effect of qTSN was associated with an earlier tillering cessation and the development of subsequent larger internodes and leaf blades and so was already visible before panicle initiation. Consequently, the panicle sink is not only generated during the reproductive phase, but appears also adjusted to the plant’s (or tiller’s) internal resources, which in turn depends on the environment. It also highlights that there is a direct response of panicle development to tiller and plant vigor that can be related, either directly to a reduction in light access and C acquisition through cloudiness or planting density, as also reported on sorghum (Lafarge et al. 2002), or indirectly to another abiotic constraint affecting C source-sink balance in the plant, for example drought (Luquet et al. 2008; Pallas et al. 2013).

Plant phenotypic plasticity is commonly observed as both plant morphology and phenology adjust to resource availability and environmental signals (Sultan 2000, Dingkuhn et al. 2005, Luquet et al. 2005), causing GxE interactions. Whatever the nature of a constraint, the plant response through adaptive plasticity ultimately modifies assimilate allocation. A relevant way to analyze processes involved in such plasticity is to directly affect plant C assimilates availability, as experienced by the plant growing under limited resource availability. In this sense, previous studies addressed the phenotypic plasticity of rice tillering, e.g., under phosphorus deficiency (Luquet et al. 2005), variable crop fertilization and population (Shimizu et al. 2010), or shading at vegetative and early reproductive stage (Lafarge et al. 2010) or grain filling (Kobata et al. 2000, Okawa et al. 2003). However, less is known on the adaptive plasticity of panicle architecture and its genetic control. A major objective of rice breeders was to develop large panicles, in terms of many fertile spikelets, in order to raise the yield ceiling, starting with the conception of new plant types with varieties having limited tillering ability (New Plant Type, NPT: Dingkuhn et al. 1991; Peng et al. 1994; Super Hybrid Rice: Peng et al. 2008; Li et al. 2014). These large panicles raised, however, questions on the adequate panicle structure and size for efficient grain filling (Peng et al. 2008) and the trade-off between panicle size and number (Dingkuhn et al. 2015).

The aim of the present study was to better quantify the variation of panicle size and architecture caused by *qTSN4* and to assess to which extent its genetic background and environmental factors affect phenotypic plasticity. For this purpose, the NILs developed by Fujita et al. (2009, 2012, 2013) and their recurrent parents, differing in panicle size, were grown into two experiments with two treatments providing differential plant access to light: a greenhouse experiment (Montpellier, France) with a full light and shading treatment during panicle development, and a field trial (IRRI, Philippines) characterized with two population densities. Panicle structure was phenotyped using detached panicles characterized by the software P-TRAP (**P**anicle **TRA**it **P**henotyping) developed by Al-Tam et al. (2013). The specific objectives were to (1) determine the plasticity of *qTSN4* phenotypes in the different environments, (2) analyze which structural traits of the panicle vary as panicle size varies, and (3) relate variation of panicle size to pre-floral stem growth rates.

## Results

### *The qTSN4* and treatment effect on yield components

Grain production per unit area (filled grain dry weight per square meter) in GH significantly increased in the presence of *qTSN4* for both genetic backgrounds (Table 2). For IR64 background, this was associated with a significant increase in filled grain number per panicle and a significant decrease in 1000 grain dry weight. For IRRI146 background, none of these yield components alone made a significant contribution to the increase in grain production. Panicle number per plant and per unit ground area was not affected by the QTL in GH.

In the field, however, *qTSN4* did not affect grain production per unit area in IR64 background and even significantly reduced it in IRRI146 background (Table 3). In IR64 background, the absence of *qTSN4* effect on grain production was associated with a reduction in panicle number (significant per unit of ground area) and in 1000-grain dry weight, which were compensated by a significant increase in the filling rate and a non-significant but numerically large increase in the number of filled grains per panicle. In IRRI146 background, the reduction in grain production in the presence of *qTSN4* was the consequence of a significant reduction in panicle number (per ground area and per plant) and filling rate, which were not compensated by the positive effects on the other yield components. The contrasting effect of *qTSN4* on grain production between the GH (positive) and field (negative or neutral) environments mainly resulted from (i) an increase in grain number per panicle that occurred in GH but not in the field, and (ii) a reduction in panicle number that occurred in the field but not in the GH.

Shading during panicle development in GH caused a significant (*P* < 0.001) reduction in grain production for both genetic backgrounds (Table 2). The reduction, averaging 47 % across genotypes, was explained by the associated reduction in 1000-grain weight (−7 %) and filled grain number per panicle (−46 %), the effects being similar among genetic backgrounds. Panicle number was not affected as shading was only imposed after panicle initiation (Table 2). No *qTSN4* x treatment interactions were observed (Table 2), indicating that QTL and shading effects acted independently. In fact, shading had been implemented after panicle initiation to avoid affecting panicle number, so that yield effects of shading would be a result of competition among panicles. Both shading and QTL effects on yield were thus mainly due to spikelet number per panicle and 1000-grain weight.

Increased plant population in the field (Table 3), which reduced access to light resources per plant but not per ground area, had no effect on grain production but strongly affected yield components. It significantly (*P* < 0.001) increased panicle number per ground area, but by compensation reduced panicle number per plant and filled grain number per panicle for all genotypes (*P* < 0.001). No *qTSN4* x treatment interactions was observed when plant density changed from 25 to 100 plants m^−2^.

The interaction between genetic backgrounds, the presence or absence of the QTL and the treatments was further analyzed with a three-stages nested design ANOVA conducted in each trial as described with P values in Additional file 1: Table S1 (electronic annex). In GH, grain production was significantly affected by shading within QTL (*P* < 0.0001) as were affected filled grain number per panicle, 1000 filled grain dry weight and filling rate in the main tiller, whereas panicle number per plant was not affected. In the field, however, grain production was not affected by plant density within QTL whereas panicle number per ground area was significantly affected (*P* < 0.0001) (Additional file 1: Table S1). This difference in performance between shading in the greenhouse and density in the field highlights an interaction between QTL and environments. A QTL effect within genetic backgrounds was observed with 1000 filled grain dry weight and filling rate for both GH and the field, but with filled grain number per panicle only in GH (Additional file 1: Table S1).

### Traits observed on individual tillers

The increase in grain production at the whole plant level caused by *qTSN4* in GH (Table 2) was also observed at the single tiller level (main culm and tiller 4) in both genetic backgrounds (Table 4). This was associated with an increase in the dry weight fraction of the panicle (compared to the stem), although not significant for T4 in IRRI146 background, and in final panicle dry matter and filling ratio (this one was not measured for tiller 4) (Table 4). In the field, the absence of *qTSN4* impact on grain production in IR64 background (Table 3) was supported by the absence of any effect at tiller level on panicle dry matter (Table 5) although the dry weight fraction (panicle/stem) was even significantly reduced in the presence of the *qTSN4*. By contrast, in IRRI146 background, the reduction in grain production (Table 3) was surprisingly associated with an increase in panicle dry matter of the main tiller but a decrease of that of tiller 4 (Table 5). This suggests a strong dominance of the main tiller over primary tillers in IRRI146 background. At the same time, dry weight fraction (panicle/stem) was not affected by *qTSN4* for both tillers.

The reduction in grain production observed at plant level due to shading in GH in both genotype backgrounds (Table 2) was associated at single tiller level with a reduced panicle dry matter and dry weight fraction (panicle/stem) in both tiller types (Table 4). In the field, the lower grain production per plant (Table 3) at high population was also confirmed at tiller level but the reduced panicle dry matter was significant only for IR64 (Table 5). No *qTSN4* x treatment effect interactions were observed at single tiller level (Tables 4 and 5).

### Traits constituting panicle architecture

**Fig. 1:**
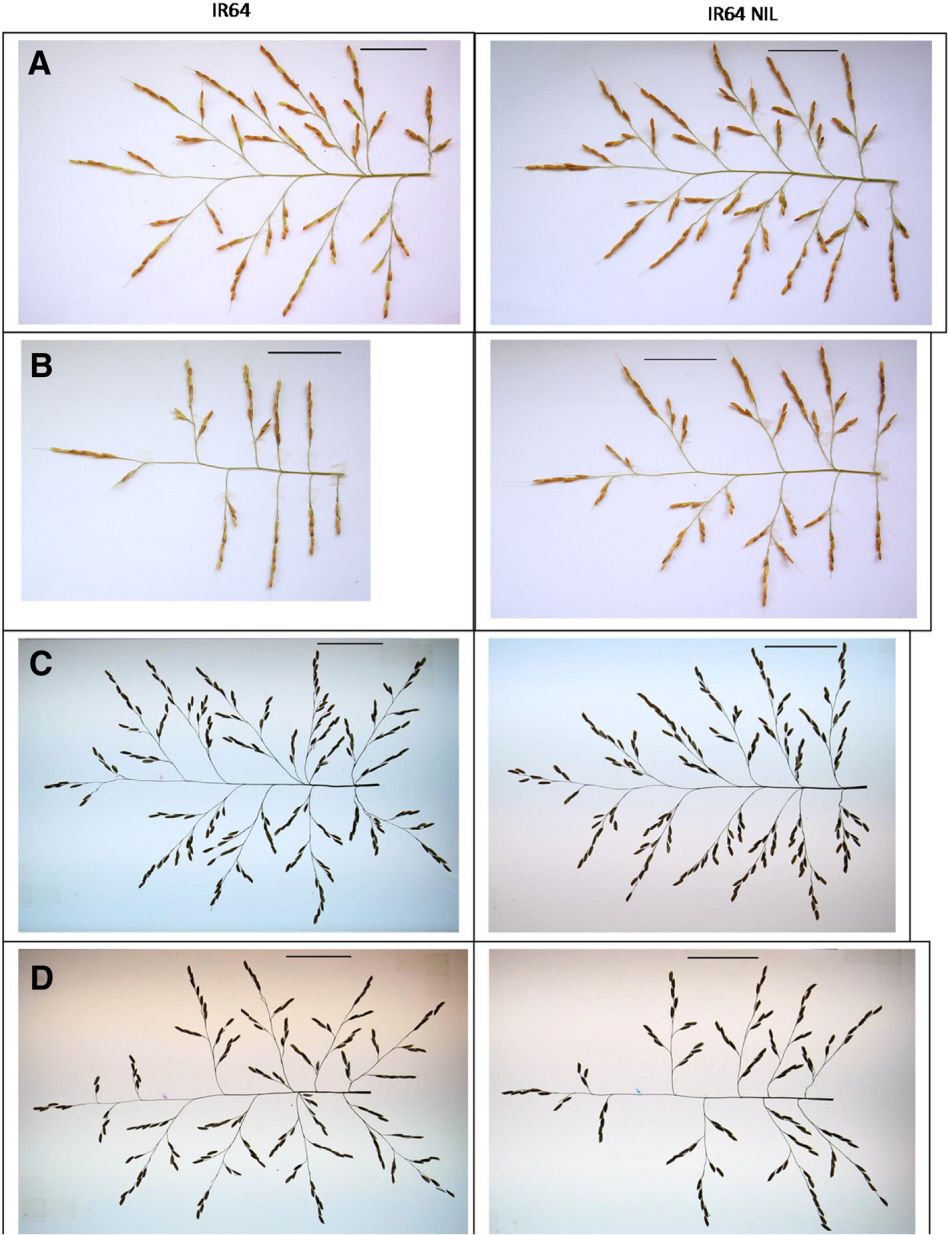
Panicle architecture analysis by P-TRAP of IR64 parent line and its NIL. **a** Greenhouse – control. **b** Greenhouse – shading. **c** Field – low density. **d** Field – high density. Scale bar 5 cm

**Fig. 2:**
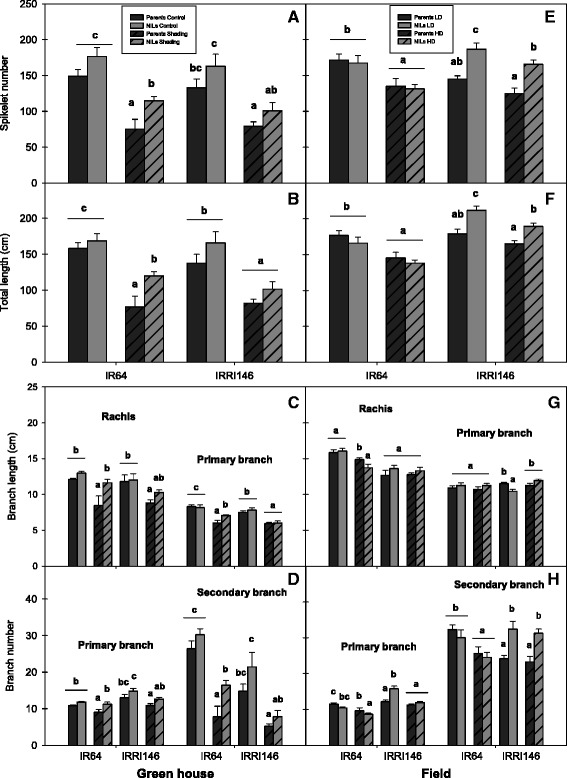
Panicle architectural traits of main tiller by PTRAP in the greenhouse under control and shading treatments (A – D), and in the field under low density (LD) and high density (HD) treatments (E – H). A and E Spikelet number per panicle. B and F Total length of branches (cm). C and G Rachis and primary branch length (cm). D and H Primary and secondary branches number. The values are mean ± SE. Different letters indicate significant differences at *P* < 0.05 according to Duncan test for multiple comparisons of each genotype (*n* = 5 for GH-CNRS, *n* = 8 for field-IRRI)

**Fig. 3:**
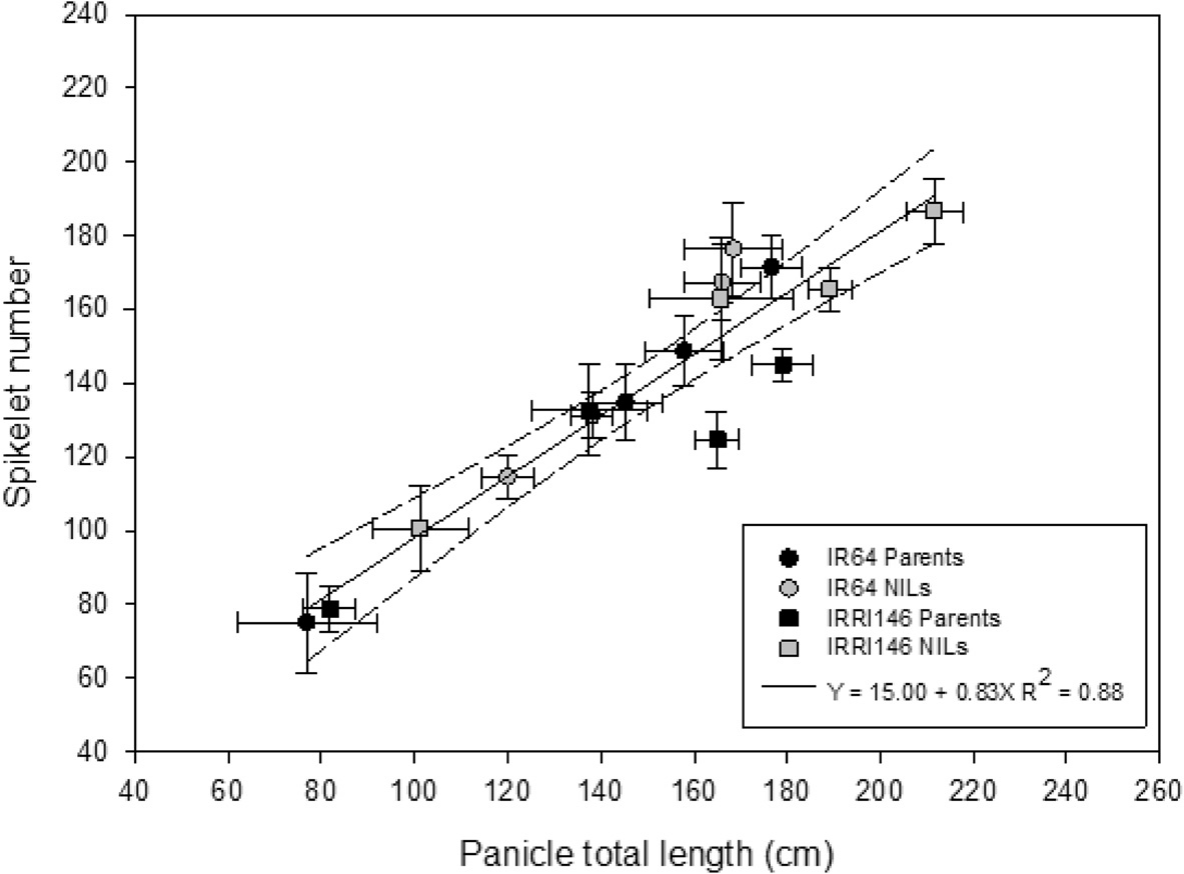
Relationships between panicle total length (cm) and spikelet number of main tiller across treatments and experiments of the parents (black symbols) and the NILs (grey symbols) of two genetic backgrounds. Regression curves are associated with confidence interval at P = 0.05 (*n* = 5 for GH-CNRS, *n* = 8 for field-IRRI)

The image processing of main-tiller panicles by the P-TRAP software resulted in the extraction of parameters related to panicle structure (panicle length, branch number) and spikelet number (as an example with IR64 background, Fig. 1). Visually the QTL effect was not discernible, and differences were only detected by measurements (as described thereafter). Regarding treatment effect, the panicle architecture of IR64 was notably modified by shading in the greenhouse and by high density in the field, while the panicle of the NIL appeared not as affected as that of the parent line. Panicle architectural traits extracted from the image collection through P-TRAP are summarized in Additional file 1: Table S2 (electronic annex) and Fig. 2. Shading in GH significantly reduced spikelet number (Fig. 2A) and total branch length (Fig. 2B) for all genotypes. The *qTSN4* numerically increased spikelet number and total length in both treatments and both genetic backgrounds but the effect was significant only for IR64 background and for the shaded treatment. Interestingly, patterns of *qTSN4* and treatment effects were similar for spikelet number and total length, both in GH and field, indicating a conserved proportionality between the number of spikelets and their supporting structure regardless of *qTSN4* and treatment effects. Consequently, a strong correlation was observed between spikelet number (SN) and total branch length (TL) across all genotypes, treatments and experiments (Fig. 3) with SN = 15.00 + 0.83TL [cm] (R^2^ = 0.88). The relative effects of *qTSN4* vs. parent on SN and TL (Additional file 1: Table S2) were also correlated (R^2^ = 0.96) and varied proportionally (Y = 0.02 + 1.07X) across the 16 combinations of background, treatment and experiment. Spikelet number and cumulative branch length were thus linked and the QTL did not affect this allometry. Consequently, spikelet density (number/branch length) varied little, although *qTSN4* increased it marginally but significantly in two cases (Additional file 1: Table S2).

The length of the rachis in GH was reduced by shading with the parents but not with the NILs (Fig. 2C), and, in the field, was not consistently modified by population treatment (Fig. 2G). Primary branch length was slightly reduced by shading in GH (Fig. 2C) but not affected by increased population in the field (Fig. 2G). No consistent positive or negative effect of *qTSN4* was observed on rachis and primary branch length. Secondary branch length was not affected by *qTSN4* in either experiment, except for a positive *qTSN4* effect observed in IRRI146 under high population in the field (data not presented). While primary branches (6–12 cm) were nearly as long as the rachis (8–16 cm), secondary branches were much shorter (2–3 cm). All branch types were longer in the field than in GH.

**Fig. 4:**
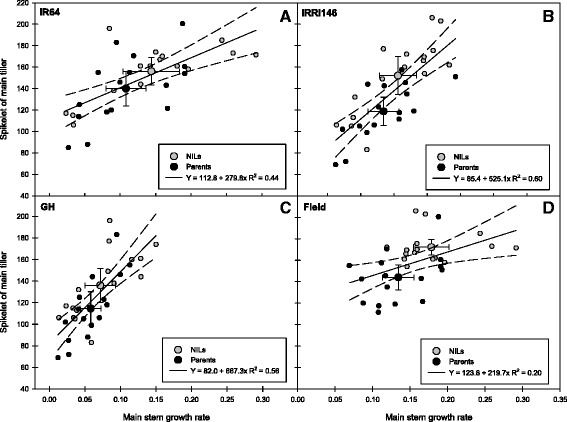
Relationships between main stem growth rate and spikelet number (SN) of main tiller of the parents (black symbols) and the NILs (grey symbols) of two genetic backgrounds. **a** IR64 background in both experiments. **b** IRRI146 background in both experiments. **c** Green house experiment in both backgrounds. **d** Field experiment in both backgrounds. Regression curves are associated with confidence interval at P = 0.05 (*n* = 16)

Patterns of *qTSN4* and treatment effects on primary branch number (Fig. 2D and G) were small and resembled those described for rachis length (Fig. 2C and G, Additional file 1: Table S2). The number of secondary branches was much more variable than that of primary branches, particularly in GH. Shading reduced it strongly (Fig. 2D), whereas increased population did so only for IR64 (both NILs). The *qTSN4* generally stimulated secondary branch number, particularly in IRRI146 (both in GH and in the field), which appeared to be the main trait of panicle plasticity. Overall, secondary branching was the main source of panicle structural plasticity, and it was significantly affected by both factors shading and QTL. The spikelet number on the main tiller was positively correlated with the main stem dry weight-based growth rate during panicle development for both genetic backgrounds (Fig. 4a and b) and both GH and field (Fig. 4c and d). In each representation, the average value of the *qTSN4 -*NILs was higher than that of the parent lines, for both spikelet number and growth rate. The correlation was stronger in GH than that in the field (Figs. 4c vs. d) and for IRRI146 than for IR64 background (Figs. 4b vs. a). On average for all experiments and treatments, the QTL increased the main stem growth rate by 38 % and SN by 15 %. Results were similar for correlations between secondary branch number and main stem growth rate (data not presented).

## Discussion

Panicle size and structure, in addition to panicle number, determine the sink components of yield potential in cereals like rice. It is known that panicle number and size are plastic traits prone to mutual compensation under competition for resources (Dingkuhn et al. 2015). The present study aimed at exploring interactions among (1) *qTSN4*, known to increase spikelet number, (2) genetic background and (3) environments involving different levels of competition for light as they affect panicle architectural traits. The choice of NILs thereby allowed us to study a single-locus effect on the behavior of panicle traits and their interactions with other traits participating into phenotype plasticity.

The study was conducted on two recipient lines (IR64, IRRI146) and their isolines (NIL) carrying *qTSN4* (syn. SPIKE), a QTL enhancing spikelet number per panicle (Fujita et al. 2009, 2012, 2013). This QTL was reported to improve, in addition to panicle size and sink capacity, assimilate source traits such as flag leaf size (Adriani et al. 2016) and carboxylation capacity (Fabre et al. 2016), owing to the association of *Nal1* gene within this QTL (Fujita et al. 2013). This might suggest that *qTSN4* effects on panicle size are not directly controlled by *qTSN4* but indirectly *via* whole-plant assimilate source-sink relations. If its effect on panicle size is indirect, we can expect it to be (1) highly prone to GxE interactions (Zhuang et al. 1997; Kobayashi et al. 2004; Chen et al. 2008), (2) non-specific with respect to panicle architectural traits (conservation of structural allometric relationships as panicle size varies), and (3) associated with pre-floral growth rate which is known to physiologically control panicle size (Horie et al. 2003; Kamiji et al. 2011). To test these hypotheses, we challenged NILs for *qTSN4* with variable light resources, focused on structural panicle traits and determined their relationship with pre-floral growth rates. The fine characterization of panicle architectural plasticity was made possible here by using an original, dedicated software P-TRAP (Al-Tam et al. 2013) and comparing i) control and shaded plants during panicle formation in a greenhouse experiment in Montpellier and ii) low and high-density crops in the field at IRRI in the Philippines.

Overall, the results confirmed a positive effect of *qTSN4* on panicle spikelet number, which in turn was correlated with the main stem growth rate during panicle development. However, in the present study, this effect suggests a dominance of the main tiller over the other tillers as it is mainly observed at main tiller level, and led to plant- and population-level yield increases only in GH but not in the field which questions the yield advantage of qTSN4 over that of parent lines IR64 and IRRI146 reported across four seasons in IRRI fields (Fujita et al. 2013).

### Hypothesis 1 – *qTSN4* phenotype depends on GxE interactions

Within each of the two experiments, no significant GxE effect was observed for any of the observed variables. In IRRI146 background, the positive *qTSN4* effect on panicle size and spikelet number per panicle was stable across environments. Fujita et al. (2013) also reported, in one growing season in the Philippines, that spikelet number per panicle was increased in IRRI146-SPIKE, the NIL backcrossed to IRRI146 background. However in this study, in IR64 background, the *qTSN4* effect was observed only in GH but it was absent in the field. This contrasts with the result reported by Fujita et al. (2013) over four seasons in the field (two trials in each the dry and the wet season) showing systematically a positive effect of *qTSN4* on panicle size and spikelet number in IR64 background. Despite some differences between studies in total N application (90 kg N in Fujita’s case *vs*. 160 kg N here) and plant density (20 plants m^−2^ with 3 seedlings per hill in Fujita’s case *vs*. 25 or 100 plants m^−2^ with 1 seedling per hill in the present case), panicle number was consistent between the two studies, being intermediate in Fujita’s case (400–525 m^−2^) compared to the values of the present study (344–362 m^−2^ under low and 471–598 m^−2^ under high population). In both field studies, *qTSN4* reduced panicle number, which was over-compensated by the increased panicle size in Fujita’s case (leading to increased yield) but under-compensated in our study. By contrast, in GH in the present study in both light treatments, *qTSN4* did not affect panicle number but increased panicle size. The interaction between *qTSN4* and the environment on plant performance was highlighted when comparing the contrasted behavior between the greenhouse and the field.

Effects of *qTSN4* on other plant morphological traits than panicle size were also observed in a variable fashion, and panicle size may not be the primary phenotypic expression of this QTL. Both panicle number and size effects may even be the results of trade-offs induced by other, phenologically earlier *qTSN4* effects. In this sense, the positive effect of *qTSN4* was also reported on blade area and internode length of the last phytomers of the tiller, associated with higher photosynthesis and starch storage in internodes (Adriani et al. 2016; Fabre et al. 2016). These results highlight the role of the tiller internal trophic status on panicle size and support the G x E interaction observed here on panicle size.

### Hypothesis 2 – *qTSN4* affects panicle size while panicle structural allometries are conserved

The panicle architectural traits that were increased in GH (together with SN) in the presence of *qTSN4* were more frequently branch number than branch length, whatever the light treatment. In the field, this was confirmed only for IRRI146 background. This is in line with Fujita et al. (2012) who observed a greater spikelet number in *qTSN4* lines, associated with an exclusive increase in panicle branch number, and with Zhang et al. (2014) who reported the increase of secondary branches, panicle length and grain number per panicle in lines carrying *qLSCHL4*, a QTL allelic to Nal1 that regulates leaf morphology and chlorophyll content. Similar observations, indicating an important role of secondary branch number in supporting panicle spikelet number, were also made for other QTLs. Mei et al. (2006), Hashida et al. (2013), Terao et al. (2010) and Ohsumi et al. (2011) reported higher spikelet number per panicle in the presence of QTL caused by more primary (*qPBN6*) or secondary branches (*qSBN1*). Interestingly, Peng et al. (2014) reported a QTL specifically for high primary branch number (*qPPB3*).

The *qTSN4* increased rachis and primary branch length in IR64 background under shade, but this effect only mitigated the length reduction induced by shading. Panicle morphogenesis starts with the formation of primary branches on successive nodes of the rachis, followed by higher-order branches (5 – 14 days after PI), and finally by floret differentiation and branch elongation (14 or 16 days after PI until heading) (Yamagishi et al. 2004; Itoh et al. 2005; Wang and Li 2005). Indeed, branch number is the main trait driving panicle sink size, and we hypothesize that light (or assimilate) resources predominantly affect numbers (of branches or spikelets) rather than their size. An analogy can be raised with the effect of shading on tillering between PI and flowering in the field, where tiller and panicle number in IR64 were mostly affected whereas stem elongation and spikelet number per panicle were comparatively conserved (Lafarge et al. 2010).

In the present study, the ratio (allometry) of spikelet number over total branch length per panicle was conserved across large variations of both variables, were they caused by *qTSN4* or experiment or treatment. The frequency of secondary branches, which provides for supplementary spikelet sites if resources allow them, was the most variable trait of panicle architecture. Panicles with increased spikelet number due to *qTSN4* thus resembled panicles receiving more resources during development, with specific structural effects being absent.

### Hypothesis 3 – *qTSN4* effect on panicle size is related to pre-floral growth rate

Increases of spikelet number per panicle caused by *qTSN4* in the field, when they occurred, were associated with reduced panicle number, probably due to competition for resources, which were also associated with earlier cessation of tillering (Adriani et al. 2016). Such physiological trade-offs in the presence of *qTSN4* were apparently also involved in the results of Fujita et al. (2013) and Okami et al. (2015) and also those of Ohsumi et al. (2011) and Weng et al. (2014) who used different QTLs affecting panicle size. The phenotype generated by *qTSN4* can thus only be understood at the whole-plant or crop level. Complex pleiotropic effects were also reported for other QTLs or genes affecting the panicle, e.g., gene *OsSPL14 (WFP*) affecting shoot branching at vegetative stage and tiller final number (Miura et al. 2010), and gene *APO1* increasing leaf number, panicle primary branch number and harvest index (Ikeda et al. 2007; Terao et al. 2010).

Cereal crop models commonly predict spikelet number or post-floral sink capacity in function of shoot growth rate during panicle development (Bouman et al. 2001; Kouressy et al. 2008). At the individual (main) tiller level, we observed a significant correlation between panicle spikelet number and stem pre-floral growth rate, which explained at least partly the effects of *qTSN4*, environment and treatment. All factor effects were thus related to the internal availability of assimilates during panicle development, which in itself is not physiologically “expensive” but may be regulated by mechanisms such as sugar signaling (Rolland et al. 2006). Both spikelet number and stem growth rates between PI and flowering were larger in the field than in GH, and the correlation across factors was particularly strong for the NILs having IRRI146 background, which also showed the more consistent *qTSN4* effect. In addition, Sheehy et al. (2001), Takai et al. (2006) and Chen et al. (2008) reported that biomass accumulation in the stem (including leaf sheath) during panicle development contributes to panicle spikelet number. Also, spikelet number shares a common genetic basis with above-ground biomass (Zhang et al. 2004) and crop growth rate at 14 days before heading (Horie et al. 2003, Kamiji et al. 2011). Similarly, the largest panicles of a sorghum plant are carried by the most vigorous tillers of the plant (Lafarge et al. 2002), indicating that panicle size depends on phenologically earlier traits associated with tiller vigor.

The pleiotropic effects of *qTSN4* may thus have a common physiological basis, although a more direct genetic control of several traits by the QTL cannot be excluded. The QTL’s primary effects shall be related to resource availability per tiller or to more upstream processes, also highlighted by Adriani et al. (2016), thereby affecting panicle size and, in some cases, yield. In this sense, according to Qi et al. (2008), the gene *Nal1* involved in leaf size, veining pattern and carboxylation is located within *qTSN4*. In addition, Takai et al. (2013) reported that *Nal1* has been identified as a QTL for GFS (Green for Photosynthesis) which changed leaf structure leading to pleiotropically enhanced photosynthesis rate. Therefore, leaf size *per se* can theoretically affect tiller number by compensation, which in turn would affect panicle number and ultimately, size.

## Conclusions

In this study, *qTSN4* was confirmed to increase panicle sink size, particularly in IRRI146 background, while proportionally stimulating panicle branching and total branch length, under different light resources. Trade-offs between panicle size and panicle number were, however, observed in most cases in this study, with a weaker effect in the field where a systematic and positive effect of *qTSN4* on grain production could not be reported. Variation in panicle sink size was related to pre-floral stem dry-weight growth, explaining some of *qTSN4*, treatment and environment effects. We conclude that *qTSN4* probably increases primarily assimilate resources available at tiller level, followed by compensatory effects on tiller/panicle number, panicle size and structure that are environment and genetic-background dependent. While the role of *qTSN4* cannot be confirmed at crop level, its explicit effect reported here at plant level was confirmed at tissue level (Fabre et al. 2016).

## Methods

### Plant materials

Two pairs of parents/near-isogenic lines (P/NIL), IR64 vs IR64 NIL, and IRRI146 vs IRRI146 NIL, were used in this study. The polymorphic locus was *qTSN4* (for total spikelet number) syn. SPIKE, associated with the gene NAL1 (Fujita et al. 2013). The NIL with IR64 background was developed by self-pollination of a plant selected from BC4F2 population. In IRRI 146 background, a whole-genome survey of 96 BC3F1 plants using 116 polymorphic SSR markers that covered all chromosomes was conducted. One BC3F1 plant was selected and self-pollinated to develop the NIL with IRRI 146 background (Fujita et al. 2013). These NILs were identified carrying the high total spikelet number (TSN) QTL between SSR markers RM3423 and RM17492 on the long arm of chromosome 4 (Fujita et al. 2012). The isoline of IR64 received this QTL from the donor parent YP9, which was derived from a cross between indica cultivar Shennung 89–366 and tropical japonica landrace Daringan. The isoline of IRRI146 received the QTL from the donor parent YP4, which was derived from a cross between tropical japonica Bali Ontjer and IRRI146 (Fujita et al. 2013). The recipient lines (parents) were chosen because of their wide adaptability: IR64 is a mega variety grown in many parts of the world (Khush, 1987) and IRRI146 is a 2nd-generation New Plant Type (NPT) variety developed at IRRI and released in the Philippines in 2007 under the name NSIC Rc158, also known as a high-yielding *indica* cultivar (Brennan and Malabayabas, 2011). The NILs were morphologically distinguishable by their larger leaf size caused by the QTL, and the plant populations were visually homogenous (no off-types).

### Design of experiment

#### Greenhouse (GH) experiment

The greenhouse experiment was performed from May to August 2013 at the National Center for Scientific Research (CNRS), Montpellier, France. The experiment adopted a two factors completely randomized design with three replications for panicle architecture, biomass and yield components measurements. The first factor was light treatment including two levels, C: natural daylight, S: shading from panicle initiation (PI) to heading. The second factor was rice genotype (G) including 4 genotypes: IR64 parent (IR64) and its NIL (*qTSN4.4* – YP9), IRRI146 parent (NSIC Rc158) and its NIL (*qTSN4.1* – YP4).

The seeds were grown in a germination chamber at 29 °C, then transplanted 4 days after germination in 3 l pots (3 seeds per pot) when seedlings were about 3 cm tall. The thinning of plant population to 1 plant per pot (downsizing to 45.65 plants m^−2^) was conducted at 4-leaf stage. Pots contained about ¾ of their volume with EGOT 140 media (17 N-10P-14 K, pH of 5) and were placed side by side (corresponding to 14.8- cm spacing) on tables filled with 5 cm water depth. Basal fertilizer was applied using Basacot 6 M+ at 2 g l^−1^, 11 N-9P-19 K +2 Mg incorporated before transplanting. A number of 104 pots were arranged on four aluminum tables, including border plants, at the beginning of the experiment. The tables were moved every week from two weeks after transplanting until maturity to avoid any bias due to the green house structure.

When the plants were at about PI (52 – 64 days after transplanting, depending on the genetic background), two layers of black net (90 × 140 cm^2^) were set up at 30 cm above the canopy enclosing 50 plants (excluding border plants). The height of the net was then adjusted based on plant growth. Two tables were dedicated to the shading treatment. The light attenuation, 58 %, was then calculated from the difference of the average of daily PAR between control and shading divided by PAR under control. The plants were exposed back to normal conditions once the plants of the same genetic background under natural light were in heading stage.

Weather data were collected from the AWS (*Automatic Weather Station*) that was installed in the center of the tables measuring Photosynthetic Active Radiation (PAR), global radiation (Rg), air temperature (T) and relative humidity (RH). The average daily air temperature throughout the crop cycle was 27.3 ± 0.6 °C. The average daily PAR for the whole crop cycle was 24.7 ± 7.1 mol m^−2^ d^−1^ under full light and 10.3 ± 3.4 mol m^−2^ d^−1^ under shading, and the average relative humidity was 66.8 ± 7.7 %.

#### Field experiment

The field experiment was performed in split plot design with four replications during the dry season (December 2013 to April 2014) at the International Rice Research Institute (IRRI) experiment station in Los Baños, Philippines (14°11’N, 121°15’E, 21 m altitude). The soil was an Andaqueptic Haplaquoll with a topsoil of 61 % clay, 32 % silt, 7 % sand pH of 6.2. The main factor was plant density, as low plant density of 20 cm × 20 cm (LD, 25 plants m^−2^), and high plant density of 10 cm × 10 cm (HD, 100 plants m^−2^). The subsidiary factor was the same rice genotypes as used in the greenhouse experiment. The density levels were chosen to reflect the recommended standard population of transplanted rice in much of the tropics including the Philippines (25 plants m^−2^) and a 4-fold greater population to approximate conditions under the increasingly popular direct seeding practice.

The seeds were soaked for 24 h, drained and incubated for another 24 h, then sown in the seeding trays in the greenhouse on December 5, 2013. The 2-week old seedlings were transplanted in the field at one plant per hill in a 2 × 2.4 m^2^ plots. The field was initially flooded to hold two puddlings and two harrowings, standing water level of 3–5 cm, was maintained as the IRRI guide field standard. Phosphorus (30 kg P ha^−1^), potassium (40 kg K ha^−1^) and zinc (5 kg Zn ha^−1^) were applied and incorporated into all the plots 2 days before transplanting. Nitrogen (60 kg N ha^−1^) was applied 1 day before transplanting, then 40 kg N ha^−1^ and 60 kg N ha^−1^ were applied at mid-tillering and panicle initiation stage, respectively.

Weather data as radiation (MJ m^−2^), daylight (hour), rainfall (mm), evaporation (mm), average temperature (°C), vapor pressure (kPa), relative humidity (%) and wind speed (m s^−1^) were collected from the IRRI meteorological station. Average daily air temperature throughout crop cycle was 25.6 ± 1.5 °C. The average daily PAR for the whole crop cycle was 31.0 ± 11.3 mol m^−2^ d^−1^, and the average relative humidity was 84.2 ± 4.8 %.

### Plant measurements

All measurements described below were performed at physiological maturity. Plants were considered at physiological maturity when 75 % of the grains of the panicles had turned yellow and the texture was in dough stage.

#### Plant phenology, morphology and biomass accumulation

The whole plant was characterized with the stem length (distance from the soil to the highest collar) of main tiller and tiller 4 (the tiller that outgrew from the bud of phytomer 4 on the main stem), the number of leaves produced by the main tiller, the total green and dead (only for greenhouse experiment) leaf number, and the green tiller number. Three consecutive plants per treatment (as three replications chosen randomly) were tagged for phenological observations in the greenhouse and in the field. In this study, we considered the ‘stem’ as the addition of internode and sheath, and the ‘tiller’ as the addition of a stem, the leaf blades, and the panicle.

Panicle initiation (PI) was determined by dissecting and observing the main tiller of randomized collected plants (border plants for field experiment) from each single treatment every second day when PI was close. PI was considered to have occurred when the first row of floral primordial was visible on the shoot apex. Flowering (FLO) was considered to have occurred within each treatment when an average of 75 % spikelets per panicle of the main tiller exerted their anthers.

Biomass accumulation at maturity was measured in GH on the plants used for phenological observation (three plants) plus two other plants. In the field, three plants used for phenology observation in both densities were chosen for biomass measurement, plus 9 additional plants in HD treatment growing next to the three initial ones, in order to get the same soil area as the LD treatment. Leaves and stems of the main tiller and tiller 4 considered separately, and of the rest of the whole plant, were characterized by weighing the materials after having dried during 72 h in an oven at 70 °C. Panicle weight of the main tiller and tiller 4 was measured after sun drying. The dry weight fraction (panicle/stem) of a single tiller was determined by calculating the dry weight of the panicle divided by the corresponding stem dry weight. Main stem growth rate during panicle development was calculated between two key dates by dividing the increase in dry matter of the main stem by the number of days between the two key dates. In GH, the main stem growth rate was calculated from PI to heading and in the field from two weeks after PI to flowering. The description of the measured plant organs with their abbreviation are described in Table 1.

**Table 1:** Description of parameters with the unit of measurement

Traits	Abbreviation	Unit
Dry weight fraction (panicle/stem) of main tiller	R_DW (panicle)_ _MT_	
Dry weight fraction (panicle/stem) of tiller 4	R_DW (panicle)__T4_	
Panicle dry weight of main tiller	DW Panicle MT	g
Panicle dry weight of tiller 4	DW Panicle T4	g
*Panicle architecture traits*:		
Spikelet number	SN	
Panicle length	PL	cm
Total length of branches	TL	cm
Rachis length	RL	cm
Averaged primary branch length	PBL	cm
Averaged secondary branch length	SBL	cm
Primary branch number	PBN	
Secondary branch number	SBN	
Spikelet density	SD	

#### Panicle architecture

Panicle architecture was analyzed by spreading the panicle of the main tiller on a white background equipped with a scale. In GH, main-tiller panicles were sampled at physiological maturity from the 5 plants used for biomass accumulation and yield components for each genotype and each treatment. In the field, main-tiller panicles for each genotype and each treatment were sampled 10 days before physiological maturity to minimize spikelet losses and sun dried before being spread over the white background. Panicles of both experiments were systematically sampled by cutting the stem just below the neck node.

**Fig. 5:**
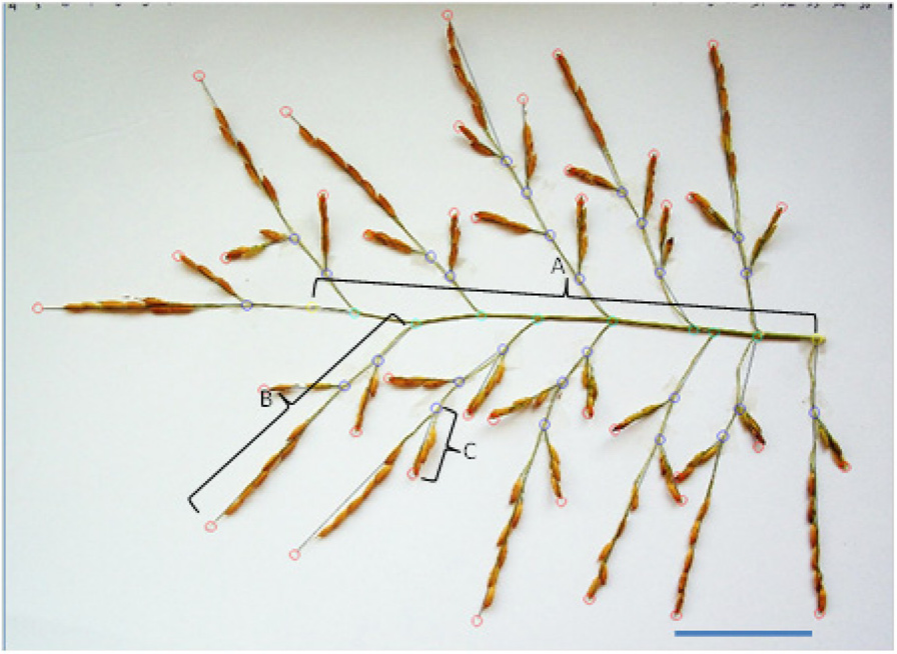
P-TRAP analysis resulting panicle structure with the rachis length (**a**), primary branch length (**b**) and secondary branch length (**c**). Scale bar 5 cm

A picture was then taken for each spread panicles to analyze its structure by using P-TRAP, a Java-based stand-alone software. This software enables to automatize the extraction of parameters such as primary, secondary and tertiary axis length and number, and spikelet number. As an example (Fig. 5), yellow circles represent the points identified by P-TRAP to mark the starting and ending nodes of a panicle primary axis, bright green circles represent the primary node starting points, and blue circles represent the starting points of secondary axes, also named the primary branches, those directly attached to the primary axes. Green circles represent the starting points of tertiary axes, also named the secondary branches, those attached to the secondary axes. Red circles represent the end of axes (Al-Tam et al. 2013).

From the image analysis, many panicle structural traits and grain traits can be obtained. For this paper, we considered rachis length (RL), average primary branch length (PBL), average secondary branch length (SBL), primary branch number (PBN), secondary branch number (SBN), and one trait describing panicle grain that is spikelet number (SN). We also determined other traits related to panicle structure like panicle length (PL) (the length of panicle from the neck node up to the tip of the last spikelet), total length (TL) of the branches (RL + (PBL × PBN) + (SBL × SBN)) and spikelet density (SD), spikelet number divided by the sum of length of all branches. The abbreviations and units of measurements are summarized in Table 1.

#### Physiological maturity and yield components

The five plants harvested at maturity in GH were separated into panicles (after taken pictures for P-TRAP analysis), green leaf blades, senescent leaves, and productive stems (culms + sheaths). In the field, we harvested all the plants within a soil base area of 0.12 m^2^ per plot that is 3 plants under LD and 12 plants under HD. They were then separated into panicles, green leaf blades, dead tissues and productive stems. The panicles were hand-threshed and then the filled spikelets were separated from the unfilled by a densitometric column (in GH) or submerging the spikelets in the water (in the field). Yield components were determined as panicle number per plant, 1000 filled grain dry weight (the grain number was counted by a grain counter in GH and manually up to 100 grain number in the field), filled grain weight and filled grain number per panicle (considering 1000 grain dry weight), filled grain weight per square meter (considering plant density). In the field, the filling rate per square meter (ratio of filled number on total spikelet number) was also determined.

### Statistical analyses

Yield components and single tiller parameters were tested for standard deviation. The graphs describing panicle architecture and main stem growth rate in relation to spikelet number were represented with standard error (standard deviation divided by square root of the number of samples). Data of Tables 2, 3, 4 and 5 and Figs. 2 and 3 were analyzed by an ANOVA procedure and mean comparison between parent vs NIL and between treatments for each pair of genotype by Duncan’s multiple range test using Microsoft® Excel 2010/XLSTAT-PRO statistical software (version 2014, Addinsoft, Inc.,Brooklyn, NY, USA). The three-stages analysis of variance was used to analyze the QTL x T interactions effect on yield components using SAS version 9.1 software (SAS Institute Inc., Cary, NC, USA). SigmaPlot® Version 11.2 software (for Windows XP and below, copyright 2009–2010), Systat Software Inc. (Chicago, IL, USA) was used for plotting data and nonlinear regressions.

**Table 2:** P-values and changes of yield component values in the presence of the QTL (in comparison to the parent) and under low access to light (in comparison to the control) of the greenhouse experiment (*n* = 5)

Parameters	IR64 background				
	QTL	Increase or reduction	Treatment	Increase or reduction	QTL x T
Filled grain dry weight (g m^−2^)	0.0003	+50	<0.0001	-47	0.582
Panicle number m^−2^	0.888	+1	0.778	+3	0.888
Panicle number plant ^−1^	0.888	+1	0.778	+3	0.888
Filled grain number panicle^−1^	0.0002	+61	<0.0001	−46	0.810
1000 filled grain dry weight (g)	0.005	−6	0.001	−9	0.196
	IRRI146 background				
	QTL	Increase or reduction	Treatment	Increase or reduction	QTL x T
Filled grain dry weight (g m^−2^)	0.007	+20	<0.0001	−45	0.37
Panicle number m^−2^	0.744	+3	0.648	+5	0.948
Panicle number plant^−1^	0.744	+3	0.648	+5	0.948
Filled grain number panicle^−1^	0.231	+12	<0.0001	−45	0.672
1000 filled grain dry weight (g)	0.038	+4	0.002	−6	0.456

**Table 3:** P-values and changes of yield component values in the presence of the QTL (in comparison to the parent) and under low access to light (in comparison to the high density) of the field experiment (*n* = 4)

	IR64 background				
Parameters	QTL	Increase or reduction	Treatment	Increase or reduction	QTL x T
Filled grain dry weight (g m^−2^)	0.612	−6	0.826	+3	0.741
Panicle number m^−2^	0.050	−15	0.0003	+51	0.131
Panicle number plant ^−1^	0.216	−10	<0.0001	−62	0.74
Filled grain number panicle^−1^	0.150	+16	0.002	−33	0.925
1000 filled grain dry weight	0.003	−6	0.802	0	0.25
Filling rate m^−2^	0.018	+22	0.208	−9	0.769
	IRRI146 background				
	QTL	Increase or reduction	Treatment	Increase or reduction	QTL x T
Filled grain dry weight (g m^−2^)	0.032	−22	0.321	−10	0.992
Panicle number m^−2^	0.010	−21	0.013	+25	0.946
Panicle number plant^−1^	0.041	−22	<0.0001	−69	0.197
Filled grain number panicle^−1^	0.774	−2	0.0003	−29	0.595
1000 filled grain dry weight	0.335	−4	0.213	+6	0.859
Filling rate m^−2^	0.017	−10	0.774	−1	0.375

**Table 4:** P-values and changes of single tiller values in the presence of the QTL (in comparison to the parent) and under low access to light (in comparison to the control) of the greenhouse experiment (*n* = 5)

	IR64 background				
Parameters	QTL	Increase or reduction	Treatment	Increase or reduction	QTL x T
R_DW (panicle)_ _MT_	0.024	+31	0.0004	−39	0.849
R_DW (panicle)__T4_	0.037	+26	0.0001	−43	0.754
DW panicle MT	0.001	+57	<0.0001	−49	0.568
DW panicle T4	0.032	+29	<0.0001	−60	0.527
Filling rate in MT	<0.0001	+47	0.086	−12	0.085
	IRRI146 background				
	QTL	Increase or reduction	Treatment	Increase or reduction	QTL x T
R_DW (panicle)_ _MT_	0.043	+24	<0.0001	−55	0.58
R_DW (panicle)__T4_	0.058	+38	<0.0001	−62	0.302
DW panicle MT	0.031	+28	<0.0001	−50	0.546
DW panicle T4	0.023	+38	<0.0001	−52	0.36
Filling rate in MT	0.059	+6	0.068	−6	0.501

**Table 5:** P-values and changes of single tiller values in the presence of the QTL (in comparison to the parent) and under low access to light (in comparison to the high density) of the field experiment (*n* = 4)

	IR64 background				
Parameters	QTL	Increase or reduction	Treatment	Increase or reduction	QTL x T
R_DW (panicle)_ _MT_	0.013	−24	0.083	−23	0.972
R_DW (panicle)__T4_	0.050	−18	0.458	−9	0.161
DW panicle MT	0.983	0	0.005	−33	0.918
DW panicle T4	0.118	−13	<0.0001	−44	0.495
	IRRI146 background				
	QTL	Increase or reduction	Treatment	Increase or reduction	QTL x T
R_DW (panicle)_ _MT_	0.215	+9	0.036	+14	0.864
R_DW (panicle)__T4_	0.569	−5	0.824	+4	0.44
DW panicle MT	0.002	+48	0.545	−7	0.811
DW panicle T4	0.969	−6	0.016	−38	0.401

## Additional file

Additional file 1: Table S1.
*P* values of yield component in the green house and field experiments. **Table S2**. The increase (positive values) and reduction (negative values) of panicle architecture traits as the result of the qTSN4 and treatment (light or density) effects. (DOCX 19 kb)
