# Supplementary material for: Rice panicle plasticity in Near Isogenic Lines carrying a QTL for larger panicle is genotype and environment dependent
Source: Rice (N Y). 2016 Jun 2;9:28. doi: 10.1186/s12284-016-0101-x (PMC4889964; doi:10.1186/s12284-016-0101-x)
Supplement: Additional file 1: Table S1. — P values of yield component in the green house and field experiments. Table S2. The increase (positive values) and reduction (negative values) of panicle architecture traits as the result of the qTSN4 and treatment (light or density) effects. (DOCX 19 kb) [file 12284_2016_101_MOESM1_ESM.docx]

**Supplement**

Table S1. P values of yield component in the green house and field experiments

| Source | Genetic background (G) | | QTL (within G) | | Treatment  (within QTL) | |
| --- | --- | --- | --- | --- | --- | --- |
|  | GH | Field | GH | Field | GH | Field |
| Filled grain dry weight (g m^-2^) | 0.0682 | 0.0734 | 0.0549 | 0.2427 | < 0.0001 | 0.9607 |
| Panicle number m^-2^ | 0.9564 | 0.0352 | 0.8698 | 0.4405 | 0.8320 | < 0.0001 |
| Panicle number plant^-1^ | 0.9564 | 0.0165 | 0.8698 | 0.2593 | 0.8320 | < 0.0001 |
| Filled grain number panicle^-1^ | 0.8000 | 0.6519 | 0.0260 | 0.2208 | < 0.0001 | < 0.0001 |
| 1000 filled grain dry weight | 0.4897 | 0.0053 | 0.0004 | 0.0033 | < 0.0001 | 0.3359 |
| Filling rate | 0.0041 | 0.0155 | 0.0001 | 0.0034 | 0.0131 | 0.7441 |

Table S2. The increase (positive values) and reduction (negative values) of panicle architecture traits as the result of the *qTSN4* and treatment (light or density) effects. Values in bold are significantly different at P<0.05 according to Duncan test for multiple comparisons of each genotype (n = 5 for GH-CNRS, n = 8 for field-IRRI).

| **GH-CNRS** | IR64 – *qTSN4* effect | | IR64 – light effect | | IRRI146 – *qTSN4* effect | | IRRI146 – light effect | |
| --- | --- | --- | --- | --- | --- | --- | --- | --- |
|  | Control | Shading | Parent | NIL | Control | Shading | Parent | NIL |
| SN | 0.186 | **0.524** | **-0.495** | **-0.350** | 0.227 | 0.273 | **-0.405** | **-0.383** |
| PL | 0.022 | **0.271** | **-0.264** | -0.085 | 0.000 | **0.137** | **-0.210** | -0.102 |
| TL | 0.067 | **0.562** | **-0.513** | **-0.288** | 0.205 | 0.238 | **-0.405** | **-0.389** |
| RL | 0.070 | **0.369** | **-0.300** | -0.104 | 0.018 | 0.168 | **-0.255** | -0.145 |
| PBL | -0.019 | **0.175** | **-0.276** | **-0.133** | 0.038 | 0.011 | **-0.211** | **-0.231** |
| SBL | -0.078 | 0.088 | **-0.222** | -0.082 | -0.047 | 0.073 | -0.097 | 0.015 |
| PBN | 0.093 | **0.244** | **-0.167** | -0.051 | 0.138 | 0.167 | **-0.169** | **-0.149** |
| SBN | 0.144 | **1.103** | **-0.704** | **-0.457** | 0.446 | 0.500 | **-0.649** | **-0.635** |
| SD | **0.111** | -0.034 | 0.051 | **-0.086** | 0.016 | 0.029 | -0.002 | 0.011 |
|  |  |  |  |  |  |  |  |  |
| **Field-IRRI** | IR64 – *qTSN4* effect | | IR64 – density effect | | IRRI146 – *qTSN4* effect | | IRRI146 – density effect | |
|  | LD | HD | Parent | NIL | LD | HD | Parent | NIL |
| SN | -0.023 | -0.027 | **-0.213** | **-0.216** | **0.286** | **0.326** | -0.1404 | **-0.114** |
| PL | **-0.045** | -0.029 | **-0.050** | -0.034 | 0.033 | **0.058** | 0.036 | 0.061 |
| TL | -0.060 | -0.049 | **-0.178** | **-0.169** | **0.182** | **0.147** | -0.079 | **-0.106** |
| RL | 0.012 | **-0.077** | -0.062 | **-0.145** | 0.074 | 0.042 | 0.008 | -0.022 |
| PBL | 0.033 | 0.050 | -0.017 | -0.001 | **-0.094** | 0.062 | -0.023 | **0.145** |
| SBL | -0.049 | **-0.072** | 0.065 | 0.040 | -0.033 | **0.214** | -0.056 | **0.184** |
| PBN | -0.098 | -0.104 | **-0.163** | **-0.169** | **0.299** | 0.056 | -0.072 | **-0.246** |
| SBN | -0.070 | -0.044 | **-0.205** | **-0.183** | **0.342** | **0.344** | -0.036 | -0.036 |
| SD | 0.040 | 0.030 | -0.047 | -0.057 | 0.079 | **0.156** | -0.073 | -0.036 |
